# Scattering Mechanisms and Suppression of Bipolar Diffusion Effect in Bi_2_Te_2.85_Se_0.15_I_x_ Compounds

**DOI:** 10.3390/ma14061564

**Published:** 2021-03-22

**Authors:** Jin Hee Kim, Song Yi Back, Jae Hyun Yun, Ho Seong Lee, Jong-Soo Rhyee

**Affiliations:** 1 Department of Applied Physics, Integrated Education Institute for Frontier Science and Technology (BK21 Four) and Institute of Natural Sciences, Kyung Hee University, Yongin 17104, Korea; song2b@khu.ac.kr (S.Y.B.); ataxtr@hanmail.net (J.H.Y.); 2 School of Materials Science and Engineering, Kyungpook National University, Daegu 41566, Korea; hs.lee@knu.ac.kr

**Keywords:** bismuth telluride, iodine doping, lattice distortion, scattering mechanism, phonon scattering

## Abstract

We investigated the anisotropic thermoelectric properties of the Bi_2_Te_2.85_Se_0.15_I_x_ (x = 0.0, 0.1, 0.3, 0.5 mol.%) compounds, synthesized by ball-milling and hot-press sintering. The electrical conductivities of the Bi_2_Te_2.85_Se_0.15_I_x_ were significantly improved by the increase of carrier concentration. The dominant electronic scattering mechanism was changed from the mixed (T ≤ 400 K) and ionization scattering (T ≥ 420 K) for pristine compound (x = 0.0) to the acoustic phonon scattering by the iodine doping. The Hall mobility was also enhanced with the increasing carrier concentration. The enhancement of Hall mobility was caused by the increase of the mean free path of the carrier from 10.8 to 17.7 nm by iodine doping, which was attributed to the reduction of point defects without the meaningful change of bandgap energy. From the electron diffraction patterns, a lattice distortion was observed in the iodine doped compounds. The modulation vector due to lattice distortion increased with increasing iodine concentration, indicating the shorter range lattice distortion in real space for the higher iodine concentration. The bipolar thermal conductivity was suppressed, and the effective masses were increased by iodine doping. It suggests that the iodine doping minimizes the ionization scattering giving rise to the suppression of the bipolar diffusion effect, due to the prohibition of the Bi_Te1_ antisite defect, and induces the lattice distortion which decreases lattice thermal conductivity, resulting in the enhancement of thermoelectric performance.

## 1. Introduction

A thermoelectric device can directly convert heat into electric energy and transfer heat by electric bias with no moving parts, no noise, and no greenhouse gas emissions. [1] Recently, because the application fields are expanding to the flexible or wearable thermoelectric devices [2], the demand for high-performance thermoelectric materials near room temperature is increasing. Thermoelectric performance mainly depends on the thermoelectric figure of merit (*zT*) which is defined by *zT* = *S^2^σT/κ*, where *S, σ, T* and *κ* are the Seebeck coefficient, electrical conductivity, absolute temperature, and total thermal conductivity, respectively [1,2].

P-type and n-type (Bi, Sb)_2_(Te, Se)_3_ are well-known high performance thermoelectric materials near room temperature. [3] The p-type bismuth tellurides show high *zT* values in the hot-deformed Bi_0.3_Sb_1.7_Te_3_ (1.3 at 380 K) [4], Te-excess Bi_0.4_Sb_1.6_Te_3_ (1.41 at 147 K) [5], melt-spun BiSbTe alloys (1.24 at 350 K [6] and 1.56 at 300 K [7]). The n-type bismuth tellurides also show relatively high *zT* values such as hot-deformed Bi_2_Te_2.3_Se_0.7_ (1.2 at 445 K) [4], Cu-doped polycrystalline Bi_2_Te_2.7_Se_0.3_ (1.10 at 373 K) [8], I-doped polycrystalline Bi_2_Te_2.7_Se_0.3_ (1.13 at 423 K) [9], CuI-doped Bi_2_Te_2.7_Se_0.3_ with hot-deformation (1.07 at 423 K) [10], textured Bi_2_Te_2.7_Se_0.3_ nano crystal (1.31 at 438 K) [11], Se-deficiency polycrystalline Bi_2_Te_2.3_Se_0.69_ (1.2 at 450 K) [12] and hot-deformed Bi_1.95_Sb_0.05_Te_2.3_Se_0.7_ (*zT* = 1.3 at 450 K) [13]. 

Even though the thermoelectric performance of n-type bismuth tellurides has been progressing significantly, the *zT* values of the n-type materials are not compatible with those of the p-type properties because the thermoelectric device’s performance is mainly determined by the average *zT* values of p-and n-type materials. [14] To reach high thermoelectric performance in n-type bismuth tellurides, there have been many studies such as the control of Se concentration Bi_2_Te_3−x_Se_x_ [4,11,15,16], Cu-doping [8,17], I-doping [9,17] Ga-doping [18], CuI-doping [10,19,20], hot-press and hot-deformation processes [8,13,21,22,23,24], etc.

Here, we investigate the iodine doping effects on the polycrystalline Bi_2_Te_2.85_Se_0.15_ compound, synthesized by the ball milling and hot press sintering. The Bi_2_Te_2.85_Se_0.15_ parent compound by zone melting method shows a high *zT* value (1.1 at 340 K) and an extremely high power factor (~5.5 mW·m^−1^·K^−2^ at 300 K) [25]. Iodine is a good n-type dopant of the bismuth tellurides, which can tune the Fermi level. It is also known that iodine doping reduces lattice thermal conductivity but the origin is not fully understood yet [9,17,25,26]. Recently, we found that the lattice distortion (or charge density wave formation) by the CuI-doping can decrease the lattice thermal conductivity with the enhancement of Hall carrier mobility [20,27]. From the electron diffraction experiment, the iodine doping in Bi_2_Te_2.85_Se_0.15_ enhances lattice distortion by the formation of the charge density wave. We propose that the lattice distortion by charge density wave (CDW) suppresses the bipolar diffusion effect and results in the enhancement of thermoelectric performance at high temperature regions. 

## 2. Materials and Methods 

The I-doped bismuth telluride (Bi_2_Te_2.85_Se_0.15_I_x_, x = 0.0, 0.1, 0.3 and 0.5 mol.%) samples were prepared by melting, ball-milling, and hot-press method. The Bi (99.999%, RNDKOREA, Gwangmyeong, Korea), Te (99.999 %, RNDKOREA, Gwangmyeong, Korea), Se (99.999%, RNDKOREA, Gwangmyeong, Korea) elements and BiI_3_ (99.999%, RNDKOREA, Gwangmyeong, Korea) powder were sealed in evacuated quartz tubes and heated at 1073 K for 24 h with slow cooling to room temperature. A planetary ball-milling pulverized the melted ingot samples for 1 h. The powders were loaded into a graphite die with an inner diameter of 12 mm and sintered by hot-press sintering at 773 K for 5 min under uniaxial pressure of 30 MPa. The relative densities of the hot-pressed samples were above 95% (7.70~7.75 g/cm^3^) comparing with the theoretical densities (8.02~8.04 g/cm^3^).

Thermoelectric properties and X-ray diffraction (XRD) patterns of the hot-pressed samples were performed with the parallel (Pa) and perpendicular (Pe) directions to the press direction. The XRD patterns were obtained using the Cu-κα radiation (D8 advance, Bruker, Germany). The temperature-dependent electrical conductivity (*σ*) and Seebeck coefficient (_S_) were simultaneously measured under the helium atmosphere by a four-point probe method using a thermoelectric properties measurement system (ZEM-3, ULVAC-RIKO, Yokohama, Kanagawa, Japan). 

The Hall carrier concentrations (*n_H_*) and the Hall coefficient (*R_H_*) were obtained by the relations of *n_H_ = −1/(R_H_e)*, where *R_H_ = ρ_xy_/H* is the Hall coefficient, *e* is the elementary charge, *ρ_xy_* is the Hall resistivity, *H* is the applied magnetic field. The Hall resistivity (*ρ_xy_*) was measured by the five-probe contact method under sweeping magnetic fields from −5 to 5 T using the physical property measurement system (PPMS Dynacool 14 T, Quantum Design, San Diego, CA, USA). The Hall mobility was obtained from the relation *μ_H_ = 1/n_H_ eρ*.

The total thermal conductivity (*κ*) was obtained by the relation of *κ = ρ_s_λC_p_*, where *ρ_s_, λ*, and *C_p_* are sample density, thermal diffusivity, and specific heat, respectively. The thermal diffusivity (*λ*) was measured by a laser flash method (LFA-457, NETZSCH, Germany). The sample density was obtained using the sample mass and dimensions. The specific heat (*C_p_*) was estimated from high temperature fitting from the measurement data by the physical property measurement system (PPMS Dynacool 14 T, Quantum Design, San Diego, CA, USA).

## 3. Results and Discussion

**Figure 1:**
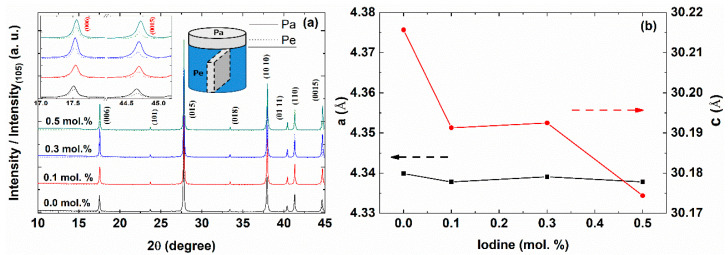
X-ray diffraction (XRD) peaks for the parallel (Pa, solid line) and perpendicular (Pe, dotted line) directions to the press direction (**a**) and the lattice parameters of the *a*-axis (black square, left axis) and *c*-axis (red circle, right axis) (**b**) of the Bi_2_Te_2.85_Se_0.15_I_x_ (x = 0.0, 0.1, 0.3, and 0.5 mol.%) compounds. The right upper inset shows the intensity ratio of the (00*l*) peaks.

Figure 1a presents the XRD patterns of the hot-pressed Bi_2_Te_2.85_Se_0.15_I_x_ (x = 0.0, 0.1, 0.3, 0.5 mol.%) compounds for two different planes of parallel (Pa, solid line) and perpendicular (Pe, dotted line) directions to the press direction, as indicated in the right inset. All the peaks were normalized with the highest (015) peaks. The XRD peaks were indexed with the Bi_2_Te_3_ structure (Rhombohedral, space group No.166) with a small change of lattice parameter.

As presented in the left-upper inset, the XRD patterns along the parallel direction showed the enhanced (00*l*) peaks, indicating that the bismuth telluride’s quintuple layers were stacked along the parallel direction to the press direction, which was similar to those of the hot-deformed Bi_2−x_Sb_x_Te_2.3_Se_0.7_ [13] and the well-aligned Bi_2_Te_3−x_Se_x_ nanocrystals [11]. The preferred orientation in polycrystal is frequently found in layer structured materials [23,28]. The anisotropic texture implies the anisotropic thermoelectric properties. 

The *c*-axis lattice parameters decreased with increasing iodine doping concentration, while the *a*-axis lattice parameter was not sensitive to the iodine concentration, as shown in Figure 1b, which was due to the smaller atomic radius of I (0.133 nm) than Te (0.137 nm). The decrease of lattice parameters was found in the Bi_2_Te_3−x_I_x_ compounds [29]. The formation energy calculation shows that the iodine can be substituted at the Te(2) sites [19]. Therefore, the reduced lattice parameters of the Bi_2_Te_2.85_Se_0.15_I_x_ (x = 0.1, 0.3, and 0.5%) compounds can be understood by the iodine substitution at the Te sites.

**Figure 2:**
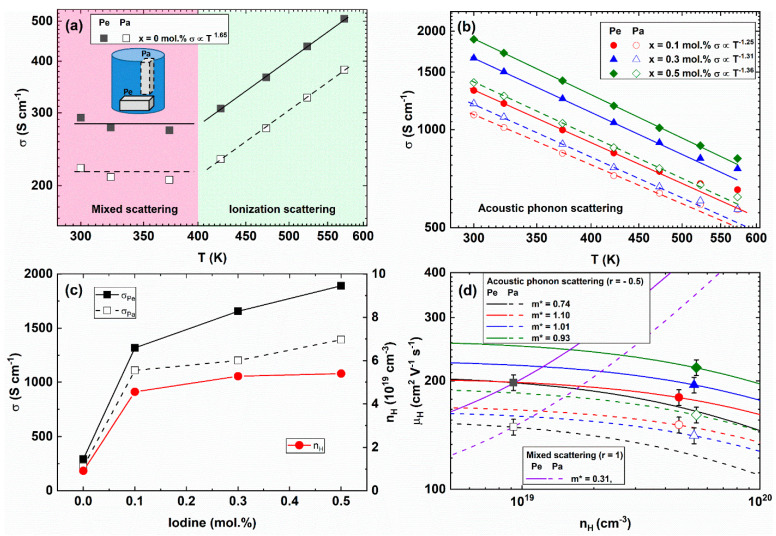
Temperature-dependent electrical conductivity *σ(T)* fitted with the mixed, ionization and acoustic scattering mechanism (lines) of the pristine (x = 0.0) compound (**a**) and iodine doped compounds (x = 0.1, 0.3, 0.5 mol.%); (**b**) Room temperature electrical conductivity (left axis, black squares) and Hall carrier concentration (right axis, red circle) as a function of the I-doping concentration (**c**) and Hall mobility with Hall carrier concentration (**d**) of the Bi_2_Te_2.85_Se_0.15_I_x_ (x = 0.0, 0.1, 0.3, 0.5 mol.%) compounds for the Pe-direction (closed symbol, solid line) and Pa-direction (open symbol, dashed line).

Figure 2a,b presents the temperature-dependent electrical conductivities *σ(T)* of the Bi_2_Te_2.85_Se_0.15_I_x_ (x = 0, 0.1, 0.3, 0.5 mol.%) compounds. The anisotropic measurement of *σ(T)* for x = 0.0 had two distinctive regions, as shown in Figure 2a; one was temperature-insensitive (T ≤ 400 K) and the other was a region linearly increasing with temperature (T ≥ 420 K). The different temperature-dependency of *σ(T)* can be understood by the distinctively different scattering mechanism. The electrical conductivity is written as *σ* = *neµ*, where mobility is implicitly represented by the effective mass m* and average relaxation time <*τ*> by *µ = e*<*τ*>/m*. The relaxation time is a function of electronic energy *E* and temperature T by the relation; $\tau =\tau_{0}{\left(E/k_{B}T\right)}^{r}$, where *r* is the scattering factor such that *r* = −1/2 for acoustic phonon scattering, *r* = 0 for a neutral impurity scattering, *r* = 1/2 for an optical phonon scattering, *r* = 1 for a mixed scattering, and *r* = 3/2 for an ionized impurity scattering [30,31,32,33]. The *σ(T)* of the pristine Bi_2_Te_2.85_Se_0.15_ compound is attributed to the mixed scattering at a lower temperature than 400 K and the ionized impurity scattering (*σ* ∝ T^1.5^) at high temperatures (T ≥ 420 K). The temperature-exponent behavior of Hall mobility may also follow the one of electrical conductivity because the carrier concentration is not sensitive to temperature in a degenerated semiconductor. So, it is reasonable to interpret the scattering exponent behavior from the temperature-dependent electrical conductivity.

The *σ(T)* of Bi_2_Te_2.85_Se_0.15_I_x_ (0.1 ≤ x ≤ 0.5 mol.%) exhibited a metallic or highly degenerated semiconducting behavior, as shown in Figure 2b. The electrical conductivity of the x = 0.5 mol.% sample (1717 S/cm at 323 K) was comparable with the one of the hot-pressed Bi_2_Te_2.85_Se_0.15_I_0.005_ compound (about 1600 S/cm at 323 K) [17]. The electrical conductivities of the Pe-direction were higher than the Pa-direction values due to the preferred orientation of the layer structure of the bismuth telluride. From the fitting of the scattering exponent, the main scattering mechanism of the Bi_2_Te_2.85_Se_0.15_I_x_ compounds could be regarded as the acoustic phonon scattering [30,34] because the scattering exponents on the I-doped compounds become close to the *σ* ∝ *T*^−1.5^, where *r* = −1.25 (x = 0.1 mol.%), −1.31 (x = 0.3 mol.%), and −1.36 (x = 0.5 mol.%).

The room-temperature electrical conductivities (left axis) were increased with increasing the iodine doping concentration for parallel (open black square) and perpendicular (closed black square) direction for the press direction, as shown in Figure 2c. The enhancement of electrical conductivity by iodine doping was mainly affected by the enhanced Hall carrier concentrations (right axis, red closed circle). From the electrical conductivity and Hall carrier concentration, the Hall mobilities *µ_H_ = 1/(neρ)* were obtained. The Hall carrier concentration (*n_H_ = n/r_H_*) and Hall mobility (*μ_H_ = μ/r_H_*) were calculated from the following equations

(1) $$\mu =\mu_{0}\frac{\left(r+\frac{3}{2}\right)F_{r\;+\;\frac{1}{2}}\left(\eta \right)}{F_{\frac{1}{2}}\left(\eta \right)}\;$$

(2) $$n=4\pi {\left(\frac{2m^{*}k_{B}T}{h^{2}}\right)}^{3/2}F_{\frac{1}{2}}\left(\eta \right)$$

(3) $$r_{H}=\frac{3}{2}\frac{\left(2r+\frac{3}{2}\right)}{{\left(r+\frac{3}{2}\right)}^{2}}\frac{F_{\frac{1}{2}}\left(\eta \right)F_{\frac{1}{2}\;+\;2r}\left(\eta \right)}{F_{r\;+\;\frac{1}{2}}{\left(\eta \right)}^{2}}$$

where m* is the effective mass of the carrier, *r_H_* is the Hall factor, *η* = *E_F_/k_B_T* is the reduced Fermi energy, and *F_n_(η)* is the *n*-th order Fermi integral given by [31,35]

(4) $$F_{n}\left(\eta \right)=\int_{0}^{\infty }\frac{x^{n}}{1+e^{x-\eta }}dx$$

The effective masses of the carrier can be obtained from the Hall carrier concentration, while the reduced Fermi energy is obtained from the Seebeck coefficient as the following equation [32,35].

(5) $$S=\pm \frac{k_{B}}{e}\left\{\frac{\left(r+\frac{5}{2}\right)F_{r\;+\;\frac{3}{2}}\left(\eta \right)}{\left(r+\frac{3}{2}\right)F_{r\;+\;\frac{1}{2}}\left(\eta \right)}-\eta \right\}$$

In the case of the acoustic phonon dominant scattering (*r* = −1/2), the Hall mobilities decreased with increasing Hall carrier concentration, as presented in Figure 2d. When we assume the mixed scattering (*r* = 1), the Hall mobility significantly increased with increasing Hall carrier concentration for perpendicular (solid black line) and parallel (black dotted line) directions, while the Hall mobilities in acoustic phonon scattering decreased with increasing Hall concentration, as shown in Figure 2d. The Hall mobilities were enhanced with increasing the I-doping concentration. The Hall mobility versus Hall carrier concentration showed that the acoustic phonon scattering was a dominant scattering mechanism.

**Figure 3:**
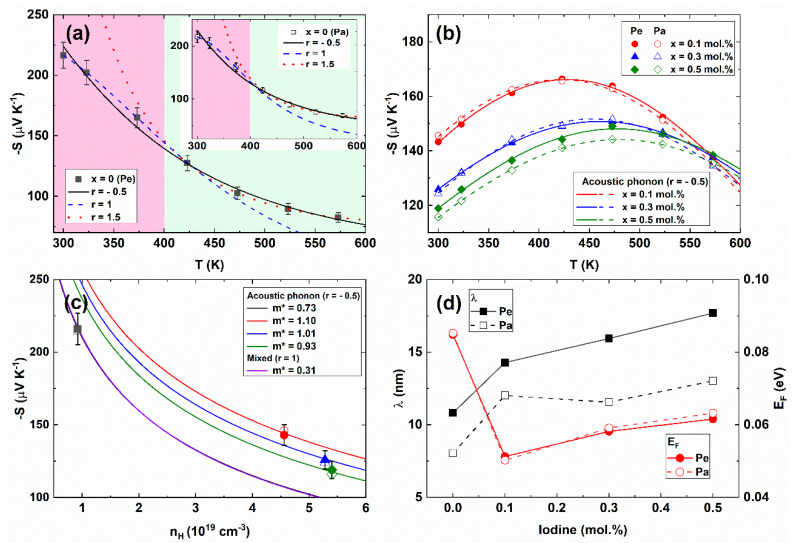
Temperature-dependent Seebeck coefficient *S(T)* of the parent compound (x = 0.0) with the mixed, ionization, and acoustic scattering mechanism (lines) (Inset is for the Pa-direction) (**a**) and for the Bi_2_Te_2.85_Se_0.15_I_x_ (x = 0.1, 0.3, 0.5 mol.%) compounds; (**b**) Seebeck coefficient S as a function of Hall carrier concentration *n_H_* for various effective masses and scattering exponents; (**c**) The carrier mean free path *λ* (left axis, black squares) and the Fermi energy *E_F_* (right axis, red circles) (**d**) as a function of I-doping concentration at 300 K for the Pe-direction (closed symbol, solid line) and Pa-direction (open symbol, dashed line).

Figure 3a,b show the temperature-dependent negative Seebeck coefficients *S(T)* for the pristine (a) and I-doped Bi_2_Te_2.85_Se_0.15_I_x_ (x = 0.1, 0.3, 0.5 mol.%) compounds (b). The negative Seebeck coefficients indicated the electronic transport of the carrier. The Seebeck coefficients of the pristine sample (x = 0.0) for the perpendicular (−216 µV/K) and parallel (−215 µV/K) directions were similar to the hot-pressed Bi_2_Te_2.85_Se_0.15_ compounds (about −220 µV/K) at room temperature [36]. The *S(T)* of the parent compound was well described by the mixed scattering exponent (*r* = 1, blue dashed line) below 425 K, while the ionization scattering (*r* = 3/2) was dominant at a high-temperature range (T ≥ 425 K). This scattering mechanism was also consistent with the *S(T)* along the Pa-direction, as presented in the inset of Figure 3a.

The temperature-dependent behavior of the Seebeck coefficient on the I-doped compounds (0.1 ≤ x ≤ 0.5 mol.%) showed a different behavior with those of the pristine compound, as shown in Figure 3b. The symbols were experimental *S(T)* data for Pe- (closed symbols) and Pa- (open symbols) directions. The solid and dashed lines were a theoretical fitting with the experiment in terms of acoustic phonon scattering (*r* = −1/2). Therefore, the dominant scattering mechanism was changed from mixed (T ≤ 425 K) and ionization scattering (T ≥ 425 K) in the pristine compound to the acoustic phonon scattering over a wide temperature range in the iodine doped compounds.

Figure 3c shows the negative Seebeck coefficient *−S* as a function of Hall carrier concentration *n_H_* at room temperature. Because the mixed (*r* = 1) and acoustic scatterings (*r* = −1/2) are dominant in parent and iodine-doped compounds, respectively, at room temperature, we used the corresponding scattering exponent within a single parabolic band assumption. The effective masses of the carriers in the I-doped samples were significantly increased (m* = 0.73 ~ 1.10, *r* = −1/2) as compared with the pristine sample (m* = 0.31, *r* = 1). The carrier effective mass could be enhanced by increasing the carrier scattering with the nanoparticles [37], resonant level formation [38], Rashba band splitting [19,39], nonparabolic band [40], the increase of band valley degeneracies [41,42] and the charge density wave (CDW) formation [43], etc. 

From the carrier mobility *μ* and the carrier effective mass m*, the mean free paths *λ* of the carrier can be calculated by the following equation, as shown in Figure 3d (left axis, black squares) [44,45].

(6) $$\lambda =\left(\frac{\mu }{e}\right)\sqrt{2E_{F}m^{*}}\;$$

(7) $$E_{F}=\left(r+\frac{3}{2}\right)\frac{\pi^{2}k_{B}^{2}T}{3eS}$$

where *E_F_* is the Fermi energy. The carrier mean free paths *λ* of the pristine sample Bi_2_Te_2.85_Se_0.15_ are about 10.8 nm (Pe-direction) and 8.0 nm Pa-direction, which are comparable with the zone melted n-type Bi_2_Te_2.79_Se_0.21_ (~7 nm) [45] and the hot-pressed CuI-doped Bi_2_Te_2.7_Se_0.3_ (10.82 nm) [10]. The mean free paths of the I-doped samples increased with increasing I-doping concentration up to 17.7 nm (Pe) and 13.0 nm (Pa). The larger *λ* in perpendicular direction than those in the parallel direction was due to the anisotropic electronic transport along with the in-plane electronic conductivity (perpendicular to the press direction).

The enhanced Hall mobilities of Bi_2_Te_2.85_Se_0.15_I_x_ were affected by the increase of carrier mean free path rather than the Fermi energy *E_F_* (right axis, red circles), as shown in Figure 3d. Because the *E_F_* subsequently decreased with the substitution of iodine concentration for x = 0.1 mol.% with the small increase of *E_F_* for high doping concentration. The carrier mean free path (*λ*) can be increased by the preferred crystallographic orientation (texturing effect) in bismuth tellurides [10] and by the reduction of defects [46]. Since the texturing effect with increasing I-doping concentration was not significantly observed in the XRD patterns, the increase of carrier mean free path could be attributed to the reduction of defects. The Bi_2_(Te, Se)_3_ compounds included the intrinsic point defects such as the Se vacancy. The Se-deficiency generated the Bi_Te_ antisite defect at the Te(Se) sites [46]. The iodine atoms substituted at the Te(Se) sites, resulting in the inhibition of the vacancy and antisite defects [19]. Therefore, the enhanced mean free path of the Bi_2_Te_2.85_Se_0.15_I_x_ compounds was attributed to the reduction of point defects.

**Figure 4:**
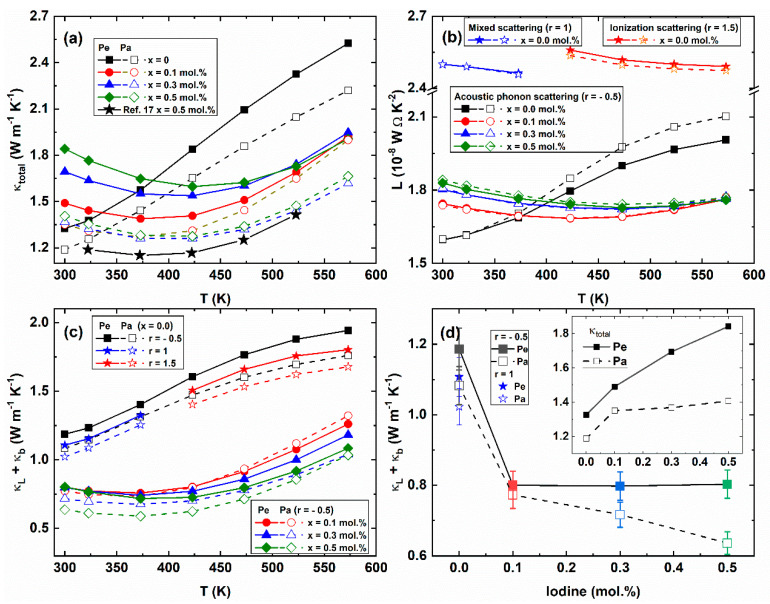
Temperature-dependent total thermal conductivity *κ_total_(T)*; (**a**) calculated Lorenz number *L(T)*; (**b**) combined lattice and bipolar thermal conductivity κ_L_ + κ_b_ of the Bi_2_Te_2.85_Se_0.15_I_x_ (x = 0.0, 0.1, 0.3, 0.5 mol.%) compounds; (**c**) Combined lattice and bipolar thermal conductivity as a function of I-doping concentration at 300 K for the Pe-direction (closed symbol, solid line) and Pa-direction (open symbol, dashed line); (**d**) Inset of (d) shows the total thermal conductivity as a function of I-doping concentration.

The temperature-dependent total thermal conductivities *κ_total_ (T)* of the Bi_2_Te_2.85_Se_0.15_I_x_ (x = 0.0, 0.1, 0.3, 0.5 mol.%) compounds are shown in Figure 4a. The *κ_total_(T)* of the pristine sample (x = 0.0) was increased with increasing temperature (black squares), which is typical behavior of bipolar diffusion effect [47]. The *κ_total_(T)* of the I-doped samples (x = 0.1, 0.3, and 0.5 mol.%) decreased with increasing temperature up to the 420 K and then increased again at high temperatures, implying the suppression of the bipolar diffusion effect by iodine doping. The total thermal conductivities of the Pe-direction were higher than those of the Pa-direction by the layer structure of the bismuth telluride [20,23].

However, in contrast with that, the Pe-direction electrical conductivities and Seebeck coefficients of the x = 0.5 mol.% sample were comparable with the data of the Bi_2_Te_2.85_Se_0.15_I_0.005_ compound [17], the thermal conductivities of the I-doped sample (0.5 mol.%) [17] were closer to the Pa-direction thermal conductivities rather than Pe-direction values of the x = 0.5 mol.% sample, which was not mention the measurement direction of the thermoelectric properties. The measurement direction matching of the power factor and thermal conductivity are necessary for the practical and correct combination of the thermoelectric properties [6]. Therefore, we believe that the thermal conductivities on this I-doped Bi_2_Te_2.85_Se_0.15_ are more reliable.

The electronic thermal conductivity can be calculated by the Wiedemann-Franz law (*κ_el_ = L_0_σT*), where *L_0_*, *σ*, and *T* are the Lorenz number, electrical conductivity, and absolute temperature, respectively. In the case of simple metals, the Lorenz number is *L_0_* = *(π^2^/3)·(k_B_/e)^2^* = 2.45 × 10^−8^ W·Ω·K^−2^. Since the Lorenz number is incorrect in a correlated metal or a degenerated semiconductor, the Lorenz number is calculated by using the following equation [31].

(8) $$L={\left(\frac{k_{B}}{e}\right)}^{2}\left(\frac{\left(r+\frac{7}{2}\right)F_{r\;+\;\frac{5}{2}}\left(\eta \right)}{\left(r+\frac{3}{2}\right)F_{r\;+\;\frac{1}{2}}\left(\eta \right)}-{\left[\frac{\left(r+\frac{5}{2}\right)F_{r\;+\;\frac{3}{2}}\left(\eta \right)}{\left(r+\frac{3}{2}\right)F_{r\;+\;\frac{1}{2}}\left(\eta \right)}\right]}^{2}\right)$$

Figure 4b presents the calculated temperature-dependent Lorenz numbers of the Bi_2_Te_2.85_Se_0.15_I_x_ (x = 0.0, 0.1, 0.3, and 0.5 mol.%) compounds. When we calculated the Lorenz number for the parent compound (x = 0.0) by mixed scattering (*r* = 1, blue star) and ionization scattering (*r* = 3/2, red star), the Lorenz numbers were about 2.4~2.5 × 10^−8^ W·Ω·K^−2^. On the other hand, for acoustic phonon scattering (*r* = − 1/2), the Lorenz numbers were in the range of (1.6 ~ 2.1) × 10^−8^ W·Ω·K^−2^. When we subtracted the electronic thermal conductivity *κ_el_* from total thermal conductivity *κ_total_*, the combined contribution of lattice *κ_L_* and bipolar thermal conductivities *κ_b_* were obtained, as shown in Figure 4c. All the samples have a bipolar diffusion effect, which is increased with increasing temperature at high temperatures (T ≥ 425 K). The bipolar diffusion effect was significantly suppressed in the I-doped Bi_2_Te_2.85_Se_0.15_I_x_ compounds (x = 0.1, 0.3, and 0.5 mol.%) even a small amount of the iodine doping. It is generally known that bismuth tellurides have a bipolar effect due to the small band gap. [48,49] The co-excitation of electron and hole in the bipolar effect decreases the Seebeck coefficient, resulting in the deterioration of thermoelectric performance. The suppression of the bipolar diffusion effect has been found in the I-doped [25,49], Cu-doped [8], Ca-doped [48], CuI-doped bismuth tellurides [19], Bi_2_Te_3−x_Se_x_ [4,25] as well as the case of an increase of energy band gap [4,25]. 

The pristine sample (x = 0.0) showed a semiconducting behavior in electrical resistivity above 400 K. From the Arrhenius equation $\sigma =\sigma_{0}e^{-E_{a}/k_{B}T}$, where *σ_0_* is a preexponential factor, *E_a_* is the activation energy, and *k_B_* is Boltzmann constant [50], the activation energy *E_a_* and band gap energy *E_g_* are obtained by *E_a_* ~ 0.076 eV and *E_g_* ~ 0.152 eV (not shown), which is estimated by the relation of the band gap energy *E_a_* ~ *E_g_*/2. In addition, the thermal band gap energy of the Bi_2_Te_2.85_Se_0.15_I_x_ (x = 0.1, 0.3, and 0.5 mol.%) compounds is obtained by about 0.14 eV from the *E_g_* = *2eS_max_T_max_* relation [25], where *S_max_* is the maximum Seebeck coefficient at the temperature (*T_max_*). The estimated band gap energies of the Bi_2_Te_2.85_Se_0.15_I_x_ (x = 0.0, 0.1, 0.3, and 0.5 mol.%) compounds are comparable with the optical band gap (*E_g_* = 0.14~0.18 eV) and thermal band gap (*E_g_* ~ 0.154 eV) of the I-doped Bi_2_(Te_1−x_Se_x_) [25] and the optical bandgap (0.14 eV) of Bi_2_Te_3−x_I_x_. [49] The obtained band gap energies showed that the band gap energy was not changed significantly by iodine doping.

The suppression of the bipolar diffusion effect by iodine doping can be described by the inhibition of the vacancy and antisite defects due to stable substitution at the Te-site. The formation energy calculation results showed that the Bi_Te1_ anti-site defects were the most energetically favorable defect in Bi-rich composition. In this case, the minority hole carriers could be generated by the Bi_Te1_ antisite defects in the n-type bismuth telluride [51]. The pristine bismuth telluride can be regarded as the Bi-rich phase due to the Te evaporation during synthesis, which is well known as the origin of the native defects [4,36]. For example, the Se-deficient compound Bi_2_Te_2.3_Se_0.7−x_ clearly showed that the bipolar effects became significant with increasing Se deficiencies [12]. The iodine substitution caused the suppression of the bipolar effect in the I-doped sample (x = 0.1, 0.3, and 0.5 mol.%) at the Te(2) sites [19]. Therefore, the reduction of point defects in the Bi_2_Te_2.85_Se_0.15_I_x_ is supported by the enhanced mean free path of the carriers. 

It is noteworthy that the lattice and bipolar thermal conductivities $\kappa_{L}+\kappa_{b}$ of the Bi_2_Te_2.85_Se_0.15_I_x_ compounds (x = 0.0, 0.1, 0.3, and 0.5 mol.%) clearly showed that the iodine doping decreased the lattice and bipolar thermal conductivities significantly, as shown in Figure 4c,d. The lattice thermal conductivity of the x = 0.1 mol.% showed the unusual anisotropic lattice thermal conductivity, which was lower lattice thermal conductivity along the Pe-direction (in-plane dominant) than that of the Pa-direction (out-of-plane dominant) values above 473 K.

The decreased lattice and bipolar thermal conductivity and the unusual anisotropic lattice thermal conductivity can be explained by the lattice distortion (or CDW). Previously, we found the lattice distortion in the hot-deformed CuI-doped Bi_2_Te_2.7_Se_0.3_ [20] and CDW-like behavior in the CuI-doped Bi_2_Te_2.1_Se_0.9_ [27]. The lattice distortion is effective to decrease lattice thermal conductivity because the lattice thermal conductivity of the in-plane direction (strong covalent bonding layer) can be smaller than the out-of-plane direction lattice thermal conductivity (weak van der Waals bonding) like a charge density wave (CDW) effect [28,52,53].

**Figure 5:**
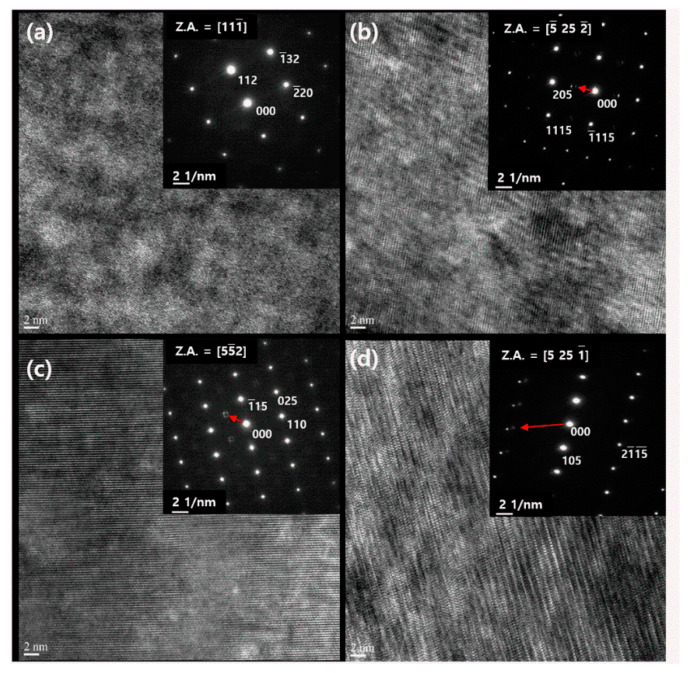
High-resolution transmission electron microscopy (HR-TEM) images and electron diffraction patterns (inset) of Bi_2_Te_2.85_Se_0.15_I_x_ compounds of x = (**a**) 0.0, (**b**) 0.1, (**c**) 0.3 and (**d**) 0.5 mol.%.

The lattice distortion of the Bi_2_Te_2.85_Se_0.15_I_x_ (x = 0.1, 0.3, and 0.5 mol.%) compounds were found in the high-resolution transmission electron microscopy (HR-TEM) and electron diffraction (ED) patterns, as shown in Figure 5. The ED pattern of the parent compound showed clear spots along the [111] axis, as presented in the inset of Figure 5a. On the other hand, there were lattice distortion peaks for the iodine doped compounds. The modulation vectors were indicated from the (000) point, marked by the red arrow. The modulation vectors increased with increasing iodine doping concentration, as shown in Figure 5b–d, indicating the shorter range lattice distortion in real space for the higher I-doping concentration. The periodic lattice modulation was also found in the CuI-doped Bi_2_Te_2.7_Se_0.3_ [20] and the CuI-doped Bi_2_Te_2.1_Se_0.9_ [27]. The lattice distortion by the iodine doping significantly decreased the lattice thermal conductivity.

**Figure 6:**
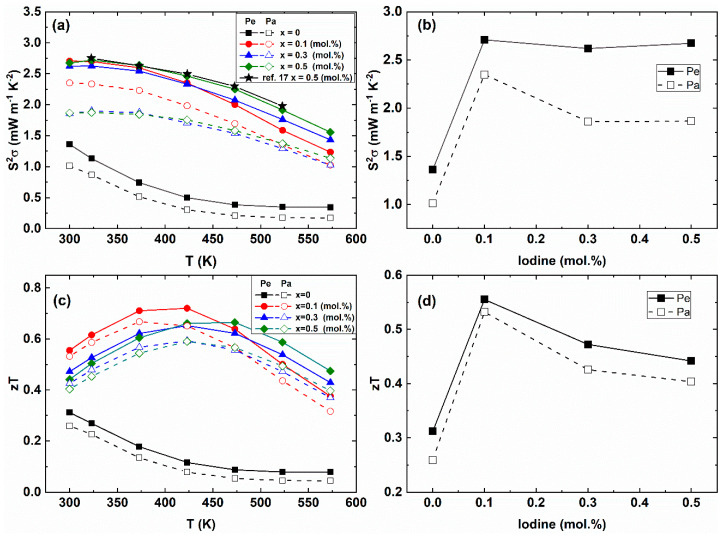
(**a**) Temperature-dependent anisotropic power factor *S^2^σ(T)*, (**b**) room-temperature power factor as a function of I-doping concentration, (**c**) temperature-dependent anisotropic *zT* values of the Bi_2_Te_2.85_Se_0.15_I_x_ (x = 0.0, 0.1, 0.3, 0.5 mol.%) compounds (**d**) and the room-temperature *zT* value with the I-doping concentration.

Temperature-dependent power factors *S^2^σ(T)* of the Bi_2_Te_2.85_Se_0.15_I_x_ (x = 0.0, 0.1, 0.3, 0.5 mol.%) compounds are presented in Figure 6a. The room-temperature power factor of the pristine sample was about 1.4 mW·m^−1^·K^−2^ for the Pe-direction (1.0 mW·m^−1^·K^−2^ for the Pa-direction). The power factors of the I-doped Bi_2_Te_2.85_Se_0.15_I_x_ (x = 0.1, 0.3 and 0.5 mol.%) compounds were increased owing to the enhancement of electrical conductivity as compared with the pristine sample. Because of the anisotropic electrical conductivity, the power factor of the Pe-direction was higher than those of the Pa-direction. Figure 6b presents that the power factor reached 2.7 mW·m^−1^·K^−2^ at 300 K for x = 0.1 mol.% for the Pe-direction. The power factor of the pristine sample (1.13 mW·m^−1^·K^−2^ at 323 K, x = 0.0) was much lower than the one of Bi_2_Te_2.85_Se_0.15_ (~2.10 mW·m^−1^·K^−2^ at 323 K) [17].

Figure 6c,d show the temperature-dependent *zT* values of the Bi_2_Te_2.85_Se_0.15_I_x_ compounds. The room-temperature *zT* values with iodine doping concentrations showed that the *zT* values of the I-doped samples (x = 0.1, 0.3, and 0.5 mol.%) were higher than those of the pristine sample (x = 0.0) by the enhanced power factor and the decreased lattice and bipolar thermal conductivity. The *zT* values of the Pe-direction were higher than the Pa-direction from the anisotropic electrical conductivity. The *zT* value of the x = 0.1 mol.% was reached 0.72 at 423 K for the Pe-direction. Therefore, the Iodine doping suppresses the bipolar diffusion effect and increases the electrical conductivity, resulting in the enhancement of the power factor.

**Figure 7:**
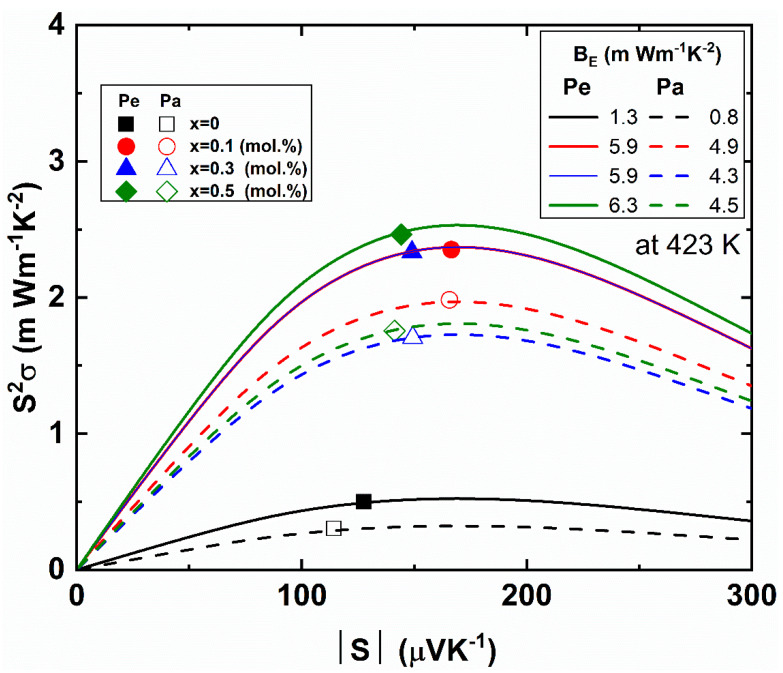
Power factor *S^2^σ* versus absolute Seebeck coefficient. Open and closed symbols are parallel (Pa) and perpendicular (Pe) directions for the pressure direction of the Bi_2_Te_2.85_Se_0.15_I_x_ (x = 0.0, 0.1, 0.3, 0.5 mol.%) compounds. Full and dashed lines are theoretically calculated power factor *S^2^σ* along the Pe and Pa directions, respectively, with respect to the estimated electronic quality factor *B_E_*, as indicated in the legend.

A recent study suggested how to find the maximum power factor without experimental optimization by defining the electronic quality factor. The electronic quality factor is defined by [54]

(9) $$B_{E}=S^{2}\sigma /\left[\frac{S_{r}^{2}\exp\left(2-S_{r}\right)}{1+exp\left[-5\left(S_{r}-1\right)\right]}+\frac{S_{r}\pi^{2}/3}{1+exp\left[5\left(S_{r}-1\right)\right]}\right]\;$$

where *S_r_* is the scaled Seebeck coefficient defined by │*S*│*e/k_B_*. Figure 7 shows the power factor *S^2^σ* with absolute Seebeck coefficient of the Bi_2_Te_2.85_Se_0.15_I_x_ (x = 0.0, 0.1, 0.3, 0.5 mol.%) compounds. From the theoretical fitting with experiment, we can estimate the electronic quality factor *B_E_*. The electronic quality factor *B_E_* values of the Iodine-doped Bi_2_Te_2.85_Se_0.15_I_x_ (x = 0.1, 0.3, 0.5 mol.%) compounds were 5.9 ~ 6.3 mW·m^−1^·K^−2^, which was significantly improved from the pristine x = 0 compound (1.3 mW·m^−1^·K^−2^) along the perpendicular direction. Because of the anisotropic thermoelectric properties, the *B_E_* values of the Pe-direction were much higher than the Pa-direction values. The higher *B_E_* values of the I-doped samples as compared to the pristine sample (x = 0.0 mol.%) clearly showed that the I-doping was effective to enhance the thermoelectric performance of the bismuth telluride.

## 4. Conclusions

In summary, we investigated the anisotropic thermoelectric properties of the Bi_2_Te_2.85_Se_0.15_I_x_ (x = 0.0, 0.1, 0.3, and 0.5 mol.%) compounds, synthesized by ball-milling and hot press sintering. The anisotropic thermoelectric properties were attributed to the structurally preferred orientation of the bismuth telluride along the (00*l*) direction with the press direction. The electrical conductivities of the I-doped samples were mainly enhanced by the increased carrier concentration. The Hall mobility was enhanced with increasing carrier concentration due to the increase of the mean free path from 10.8 to 17.7 nm by iodine doping. The enhanced mean free path was caused by the reduction of point defects. We found that the carrier scattering mechanism was changed from the mixed (T ≤ 400 K) and ionization scattering (T ≥ 420 K) for pristine compound (x = 0.0) to the acoustic phonon scattering for iodine doped compounds (x = 0.1~0.5 mol.%). The electron diffraction results clearly showed that the I-doping generated the periodic lattice distortion in the bismuth telluride. For increasing I-doping concentration, the modulation vector in momentum space was increased, in other words, the shorter range of lattice distortion for the higher iodine doping concentration. The periodic lattice distortion could increase the carrier effective mass as the CDW effect. The ionization scattering of the carrier should be minimized to suppress the bipolar diffusion effect in thermal conductivity due to the prohibition of vacancy and antisite defects due to stable substitution at the Te-site, while the lattice distortion decreases the lattice thermal conductivity by iodine doping. Because of the enhanced power factor and the decreased lattice and bipolar thermal conductivity by the iodine doping, we observed the enhancement of *zT* values from 0.31 (x = 0.0) to 0.56 (x = 0.1 mol.%) at 300 K (0.72 at 423 K for Pe-direction). This research strongly suggests that the minimization of ionization scattering suppresses the bipolar diffusion effect by hindering vacancy and defect states, and lattice distortion decreases lattice thermal conductivity, resulting in the enhancement of thermoelectric performance.

## Data Availability

The data presented in this study are available on request from the corresponding author.
